# Case report: extreme coronary calcifications and hypomagnesemia in a patient with a 17q12 deletion involving *HNF1B*

**DOI:** 10.1186/s12882-019-1533-5

**Published:** 2019-09-09

**Authors:** Howard J. Li, Catherine Groden, Melanie P. Hoenig, Evan C. Ray, Carlos R. Ferreira, Willam Gahl, Danica Novacic

**Affiliations:** 1 Harvard Medical School, Boston, MA 02115 USA; 2 National Institute of Mental Health, NIH, Bethesda, MD 20892 USA; 3 Undiagnosed Diseases Program, Office of the Clinical Director and National Human Genome Research Institute, NIH, Bethesda, MD 20892 USA; 4 Division of Nephrology, Beth Israel Deaconess Medical Center, Boston, MA 02215 USA; 5 Renal-Electrolyte Division, Department of Medicine, University of Pittsburgh, Pittsburgh, PA 15261 USA; 6 Medical Genetics Branch, National Human Genome Research Institute, NIH, Bethesda, MD USA

**Keywords:** 17q12 deletion syndrome, *HNF1B*, Hypomagnesemia, Hyperparathyroidism, Vascular calcification, Case report

## Abstract

**Background:**

17q12 deletion syndrome encompasses a broad constellation of clinical phenotypes, including renal magnesium wasting, maturity-onset diabetes of the young (MODY), renal cysts, genitourinary malformations, and neuropsychiatric illness. Manifestations outside of the renal, endocrine, and nervous systems have not been well described.

**Case presentation:**

We report a 62-year-old male referred to the Undiagnosed Diseases Program (UDP) at the National Institutes of Health (NIH) who presented with persistent hypermagnesiuric hypomagnesemia and was found to have a 17q12 deletion. The patient exhibited several known manifestations of the syndrome, including severe hypomagnesemia, renal cysts, diabetes and cognitive deficits. Coronary CT revealed extensive coronary calcifications, with a coronary artery calcification score of 12,427. Vascular calcifications have not been previously reported in this condition. We describe several physiologic mechanisms and a review of literature to support the expansion of the 17q12 deletion syndrome to include vascular calcification.

**Conclusion:**

Extensive coronary and vascular calcifications may be an extension of the 17q12 deletion phenotype, particularly if hypomagnesemia and hyperparathyroidism are prevalent. In patients with 17q12 deletions involving *HNF1B*, hyperparathyroidism and hypomagnesemia may contribute to significant cardiovascular risk.

## Background

17q12 deletions involving *HNF1B* are associated with maturity-onset diabetes of the young type 5 (MODY5) and abnormalities in renal structure and function, including congenital malformations of the kidney and ureter, renal cysts, electrolyte abnormalities, and renal failure. Many of the renal and endocrine abnormalities, including hypomagnesemia, have been attributed to the involvement of *HNF1B*, a major transcription factor encoded within the 17q12 region. Further reports showed that 17q12 deletion is associated with neuropsychiatric manifestations such as developmental delay, autism spectrum disorder, and schizophrenia. Cardiovascular manifestations have not been described.

## Case presentation

A 62-year-old Caucasian male was referred to the NIH Undiagnosed Diseases Program (UDP) by his nephrologist in January 2018 and enrolled in protocol 15-HG-0130, “Clinical and Genetic Evaluation of Individuals With Undiagnosed Disorders Through the Undiagnosed Diseases Network” [1].

The patient’s hypomagnesemia came to clinical attention at age 45. One week after starting triamterene-hydrochlorothiazide for hypertension, he complained of profound malaise, myalgia, and arthralgia and was found to have severe electrolyte abnormalities: hypomagnesemia, hypocalcemia, and hypokalemia (Table 1). Aside from his 1 week trial of triamterene-hydrochlorothiazide, the patient had not taken any other diuretics. The patient was hospitalized for electrolyte repletion and evaluation. Since then, he required aggressive oral and intravenous magnesium supplementation daily (magnesium lactate, 504 mg p.o., magnesium sulfate, 5 g i.v.), along with amiloride 10 mg tid for magnesium sparing. Laboratory monitoring has been consistently notable for renal magnesium wasting and hyperparathyroidism, but otherwise normal renal function.

**Table 1:** Representative laboratory values (August 2000 – January 2018)

	Aug. 2000^a^	Aug. 2000^b^	Jun. 2009	May 2017	Jan. 2018^c^
Na (136–145 mEq/L)	**133**	140	140	139	143
K (3.5–5.0 mEq/L)	**2.8**	4.1	4.3	4.3	4.2
Cl (98–106 mEq/L)	102	101	102	101	101
CO2 (23–28 mEq/L)	28	29	28	26	23
BUN (8–20 mg/dL)	12	17	17	16	20
Cr (0.7–1.5 mg/dL)	1.0	1.0	0.9	0.7	0.9
eGFR (CKD-EPI) (> 90 mL/min/1.73m^2^)	90	90	96	102	91
Uric acid (3.7–8.6 mg/dL)					7.0
HbA1c (4.0–5.6%)		5.6	6.4	5.8	5.6
PTH (10–65 pg/mL)		**80**		**184.4**	**148.6**
Ca (8.6–10.2 mg/dL)	**7.7**	9.2	9	9.1	9.3
Phos (3.0–4.5 mg/dL)		4.4	2.9	3.9	3.0
25-OH Vit D (20–60 ng/mL)			25	22.7	25
Mg (1.6–2.6 mEq/L)	**1.0**	**1.1**	**1.5**	**1.4**	1.6
24 h Urine Mg (14–290 mg/24 h)		**595**	**322**	**537**	**553**
24 h Urine Ca (50–300 mg/24 h)		**326**		294	**354**
24 h Urine Cr (1.00–2.00 g/24 h)		1.60		1.27	1.13

Relevant values in bold

^a^Initial ED Presentation

^b^Clinically stable, on discharge

^c^NIH evaluation

At age 61 he developed unstable angina and was treated with a coronary artery bypass graft in 2016. At that time, diabetes mellitus type 2 and hyperlipidemia were also noted and well-controlled with medication. In addition to magnesium supplementation, the patient’s medications were aspirin (81 mg qd), amiloride (10 mg tid), furosemide (40 mg qd), amlodipine (10 mg qd), carvedilol (6.25 mg bid), metformin (500 mg bid), glimepiride (4 mg qam), ezetimibe (10 mg qd), and simvastatin (40 mg qd). He did not take any proton-pump inhibitors.

Family history was significant for sudden cardiac death (maternal grandfather, deceased, age 40), ruptured aortic aneurysm (father, deceased, age 79), pancreatic cancer (mother, deceased, age 77), and mild intellectual disability with speech delay (son, age 28). There was no family history of hypomagnesemia. There was no cosangunity.

The patient appeared well but obese (BMI 35.2). Mental status was suspicious for subtle cognitive and social deficits. The remainder of the physical exam, including vital signs, cardiopulmonary exam, and reflexes, were normal.

Laboratory evaluation at the NIH in January 2018 was significant for hyperparathyroidism and increased urinary magnesium excretion with an inappropriately high fractional excretion of magnesium of 23% based on 24-h urine collection (normal < 4%), and slightly increased urinary calcium excretion. Serum electrolytes were normal while receiving oral and intravenous magnesium repletion at the time of NIH evaluation. Aside from magnesium, urine electrolytes were normal (Table 1). A comprehensive metabolic panel including liver and lipid studies was unremarkable.

**Fig. 1:**
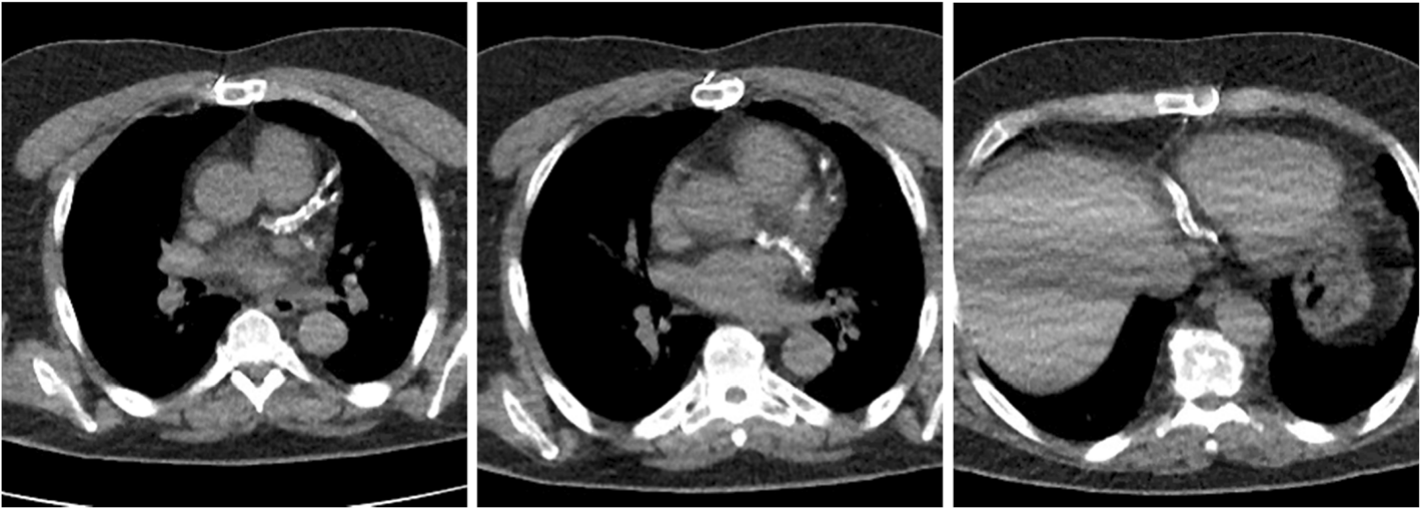
Axial CT images depicting extensive calcifation of the left anterior descending (*left*), left circumflex (*middle*), and distal right and posterior descending (*right*) coronary arteries

Renal ultrasound showed multiple cortical cysts, bilaterally. Bone density scan showed osteopenia at the left forearm (T = − 2.0) and lumbar spine (T = − 1.9). CT scan showed vascular calcifications of the anterior and posterior cerebral arteries, bilateral carotid arteries, and calcific spots along the descending aorta and iliac arteries. Notably, there was extensive calcification of the coronary arteries (Fig. 1), with a coronary artery calcium (CAC) score of 12,427 by the Agatston scoring method [2]. Echocardiogram was normal. Neuropsychiatric testing revealed significant abnormalities in short term memory despite adequate attention, language and reasoning skills. The patient also had a deficit in fine motor speed but not in processing speed. Prior to referral to the NIH, the patient received genetic testing and was found to have a 17q12 deletion of 1.5 megabases via chromosomal microarray. Whole genome sequencing confirmed a 17q21 deletion (ch17:34,815,551-36,223,325) involving 15 genes including *HNF1B*, and did not reveal any variants known to be associated with vascular calcification. Genetic counseling was offered, and family members did not elect for genetic testing at the time of evaluation.

## Discussion and conclusion

### Phenotypic features of 17q12 deletion involving *HNF1B*

17q12 deletion syndrome encompasses a broad range of phenotypes, including diabetes mellitus, renal malformations, impaired renal function, and neuropsychiatric disorders [3]. Hepatocyte Nuclear Factor 1B (*HNF1B*) is a critical transcription factor gene within the 17q12 deletion region. While *HNF1B* polymorphisms have been known to cause hereditary Maturity-Onset Diabetes of the Young (MODY), *HNF1B* deletion now appears to be responsible for many other manifestations of 17q12 deletion syndrome [4]. *HNF1B* is critical to pancreatic development and beta cell function, a mechanism linking *HNF1B* polymorphisms to MODY and neonatal diabetes [4, 5]. Recent studies also documented hyperparathyroidism in patients carrying *HNF1B* mutations and identified it as a regulator of PTH expression, suggesting that *HNF1B* deficiency causes hyperparathyroidism independent of associated renal failure [6]. Renal cysts (most common), renal hypoplasia or agenesis, and genital malformations have also been noted, consistent with *HNF1B*’s involvement in kidney and urinary tract morphogenesis [7]. Neuropsychiatric manifestations of 17q12 deletion syndrome may be independent from *HNF1B* deletion [8], although this is controversial [9]. Hypomagnesemia is a common manifestation of *HNF1B* mutations [10]. *HNF1B* is essential for expression of *FXYD2*, a subunit of the sodium-potassium ATPase critical for driving transcellular magnesium transport in the kidney’s distal convoluted tubule (DCT). Mutations in both *HNF1B* and *FXYD2* are associated with renal magnesium wasting [6, 10]. While the inheritance of these renal syndromes have been described as autosomal dominant traits, deleterious *HNF1B* polymorphisms frequently arise from de novo mutations. Gitelman syndrome (GS) is often initially considered in patients with *HNF1B*-related renal disease who present with hypomagnesemia; this was the case with our patient before GS was excluded by the absence of hypocalciuria and by genetic testing.

Findings in this patient consistent with known manifestations of 17q12 deletion involving *HNF1B* include multiple renal cysts, diabetes mellitus, hypomagnesemia, hyperparathyroidism, and cognitive deficits. This is the first description of extreme coronary calcifications in a patient carrying a 17q12 deletion.

### Hypomagnesemia

Hypomagnesemia may be attributed to three etiologic categories: redistribution, decreased gastrointestinal absorption, and increased renal excretion. Redistribution occurs when magnesium is sequestered in specific tissues or compartments, such as in refeeding syndrome and fat saponification in acute pancreatitis. Causes of decreased gastrointestinal uptake include malnutrition, malabsorption, and diarrhea. Proton pump inhibitors commonly cause hypomagnesemia by altering the pH of gastric secretions and limiting magnesium absorption by enterocytes. Increased renal excretion can be due to excessive diuresis, medications (particularly loop and thiazide diuretics), acquired tubular dysfunction, and more rarely, genetic causes such as Gitelman and Bartter syndromes and defects of tubular components such as CaSR, claudins 19 and 14, among others. Quantifying renal magnesium excretion via a 24-h urine collection for fractional excretion calculation is the most definitive test when renal magnesium loss is suspected.

This patient presented in late adulthood within 1 week of initiating a thiazide diuretic. The potential side effects of thiazide diuretics are well-characterized and include hypomagnesemia. Specifically, the DCT is a target site for both therapeutic action (thiazide-sensitive Na/Cl symporter antagonism) and toxic effects (apoptosis of DCT epithelial cells, decreased expression of DCT-specific magnesium transporters) [11]. The DCT is also a key tubular segment for regulated, transcellular magnesium transport, and a segment where *HNF1B* haploinsufficiency contributes to hypomagnesemia [10, 12], though the potential effect of *HNF1B* deletion on other nephron segments and extra-renal determinants of magnesium balance cannot be excluded. This patient likely had longstanding but subclinical *HNF1B*-related renal magnesium wasting with a heightened genetic vulnerability to thiazide diuretics, which may explain his initial presentation shortly after initiating a thiazide diuretic.

Decades after discontinuing the causal agent, however, this patient was unable to reduce his supplementation regimen without developing symptomatic hypomagnesemia. One explanation is that acute nephrotoxic episodes result in permanent but subclinical loss of tubular function. In the setting of preexisting *HNF1B* haploinsufficiency and impaired magnesium transport at the DCT, this may result in permanent, clinically significant magnesium wasting. A comparison can be made with cisplatin, an agent known to also cause irreversible tubular dysfunction and renal magnesium wasting due to specific nephrotoxic effects on the DCT [13]. Additionally, manifestations of *HNF1B* haploinsufficiency may worsen with age, as both childhood and late-adulthood presentations of HNF1B-related disease have been described [14]. Variability in *HNF1B*-related hypomagnesemia may be influenced by the extent of 17q12 deletion (1.5 megabases involving 15 genes in this patient), the effect of metabolic and cardiovascular comorbidities (obesity, hypertension, and diabetes), and the presence of structural renal disease (renal cysts).

### Coronary calcification as a possible physiologic sequela of *HNF1B* deletion

With a coronary arterial calcium (CAC) score of 12,427, the extent of this patient’s coronary calcification is unprecedented. While this patient has several cardiovascular risk factors (history of unstable angina, obesity, diabetes, hypertension, and hyperlipidemia), one study found that among individuals in the United States with five or more risk factors for coronary artery disease (obesity, diabetes, hypercholesterolemia, hypertension, smoking, sedentary lifestyle, or family history), the mean CAC score was 271.5 [15]. In another study, the mean CAC score among 92 patients with a history of myocardial infarction was 427.0 ± 516.2 [16]. Despite the considerable variability in CAC scores across studies and populations, this patient’s CAC score is 1–2 orders of magnitude outside the typical CAC score distribution for both low and high CAD-risk groups. We review three overlapping mechanisms that may link this patient’s *HNF1B* deletion to his extensive vascular calcifications:

1. Bone-vascular axis dysregulation*.* The relationship between skeletal and vascular mineralization is known as the bone-vascular axis [17]. A major mechanistic underpinning of the bone-vascular axis is the fact that calcification of bone and vasculature share common regulators, most notably parathyroid hormone (PTH). *HNF1B* is required for repression of PTH transcription, and *HNF1B* haploinsufficiency leads to primary hyperparathyroidism [6]. Also, while severe hypomagnesemia potently blocks PTH secretion, moderate hypomagnesemia is a stimulator of PTH [18]. With the exception of his initial presentation of severe magnesium deficiency and subsequent hypocalcemia, this patient’s parathyroid physiology while on magnesium supplementation may be best described as primary hyperparathyroidism due to *HNF1B* deletion, exacerbated by moderate hypomagnesemia. This patient’s hyperparathyroidism, osteopenia, and vascular calcifications constitute a clinical triad similar to chronic kidney disease–mineral bone disorder (CKD-MBD) [19]. CKD-MBD entails a complex pathophysiological model, in which hyperphosphatemia, secondary hyperparathyroidism, inflammatory cytokines, and oxidized lipids all contribute to vascular calcification. Although renal insufficiency is essential to CKD-MBD, the independent contribution of hyperparathyroidism to bone-vascular axis dysregulation is relevant to this case. In animal models, PTH induces bone turnover and arterial calcification, even in the absence of hypercalcemia, hyperphosphatemia, or renal insufficiency [20]. Finally, epidemiologic studies confirm that bone density is inversely correlated with vascular calcification, independent of age, in patients both with and without chronic kidney disease [21, 22].
2. Hypomagnesemia and increased calcium crystal precipitation. The association between hypomagnesemia and vascular calcification is well-studied. Magnesium directly inhibits calcium-phosphate crystal formation in vitro [23], while magnesium supplementation in an animal model for a heritable disorder of vascular calcification prevented mineralization [24]. Large population studies show that magnesium intake in people free of cardiovascular disease was inversely associated with coronary artery calcification [25], whereas serum magnesium concentrations are inversely associated with CAC both in a general healthy population [26–28], as well as in patients with chronic kidney disease [29]. Low magnesium levels may decrease expression of endogenous inhibitors of calcification, notably matrix Gla-protein (MGP) and fetuin-A, which sequester calcium and phosphate ions, act as chaperones for calcium-phosphate complexes, and inhibit crystal nucleation at the extracellular matrix [23, 30, 31].
3. Cell-mediated osteogenic activity. Hypomagnesemia and hyperparathyroidism modulate osteogenic activity in endothelial and vascular smooth muscle cells (VSMC), a major mechanism of arterial calcification, especially in atherosclerosis. In endothelial cells, low magnesium levels increase atherosclerotic signaling: ROS production, NF-kB activation, and cytokine release [32, 33]. Magnesium deficiency also promotes endothelial permeability allowing for invasion of LDL particles, macrophages, and VSMCs, a key initiating step in atherosclerosis and vascular calcification [32]. In VSMCs, magnesium suppresses transcription of potent osteogenic genes, including BMP2, RUNX2, and osteocalcin [23]. Similarly, elevated PTH levels stimulate osteogenic signaling in endothelial cells, including NF-kB-dependent upregulation of BMP2 [34]. In VSMCs, hyperparathyroidism ultimately promotes calcification, even though the direct effect of PTH and PTH-related peptide (PTHrP) on VSMCs appears to be one of inhibited calcification [17]. It is thought that longstanding hyperparathyroidism causes receptor internalization and antagonism via excess PTH fragments, paradoxically reducing the protective effects of PTH signaling at VSMCs [35]. Furthermore, fetuin-A and MGP, in addition to directly inhibiting calcium crystal formation as discussed above, regulate VSMCs; both hypomagnesemia and hyperparathyroidism decrease fetuin-A and MGP levels, which in turn promote the osteogenic phenotype of VSMCs [30, 31]. Finally, *HNF1B* may be a transcription factor for *SPP1*, also known as osteopontin [36]. Osteopontin is a secreted protein with multiple functions, including regulating soft tissue mineralization. Particularly in VSMCs, osteopontin inhibits hydroxyapatite crystal deposition and calcification. Decreased expression of osteopontin due to haploinsufficiency of *HNF1B* may be another mechanism promoting vascular calcification [37].

In conclusion, vascular calcifications have not been previously described in 17q12 deletion syndrome. Based on our discussion of this patient’s findings, review of literature, and pertinent physiologic mechanisms, vascular calcifications are a plausible extension of the 17q12 deletion phenotype and may contribute significantly to the cardiovascular risk of affected patients, particularly in the setting of hypomagnesemia and hyperparathyroidism. Evaluation, surveillance, and aggressive management of cardiovascular risk factors should be considered for individuals with 17q12 deletion syndrome.

## Data Availability

The datasets used and/or analysed during the current study available from the corresponding author on reasonable request. Clinical phenotyping deposited into the PhenomeCentral database via PhenoTips.

## Abbreviations

CAC: Coronary Artery Calcium [Score]
CKD-MBD: Chronic kidney disease–mineral bone disorder
DCT: Distal convoluted tubule
ESRD: End Stage Renal Disease
*HNF1B*: Hepatocyte Nuclear Factor 1B
LDL: Low Density Lipoprotein
MGP: Matrix Gla protein
MODY: Maturity-onset diabetes of the young
PTH: Parathyroid hormone
PTHrP: Parathyroid Hormone Related Peptide
ROS: Reactive Oxygen Species
VSMC: Vascular smooth muscle cell
